# IMU-to-Segment Assignment and Orientation Alignment for the Lower Body Using Deep Learning

**DOI:** 10.3390/s18010302

**Published:** 2018-01-19

**Authors:** Tobias Zimmermann, Bertram Taetz, Gabriele Bleser

**Affiliations:** 1 Junior Research Group wearHEALTH, University of Kaiserslautern, Gottlieb-Daimler-Str. 48, 67663 Kaiserslautern, Germany; tobias.zimmermann@cs.uni-kl.de (T.Z.); bleser@cs.uni-kl.de (G.B.); 2 Augmented Vision Department, DFKI, Trippstadter Str. 122, 67663 Kaiserslautern, Germany

**Keywords:** inertial sensors, automatic sensor placement, automatic sensor alignment, neural networks, deep learning, LSTM, CNN

## Abstract

Human body motion analysis based on wearable inertial measurement units (IMUs) receives a lot of attention from both the research community and the and industrial community. This is due to the significant role in, for instance, mobile health systems, sports and human computer interaction. In sensor based activity recognition, one of the major issues for obtaining reliable results is the sensor placement/assignment on the body. For inertial motion capture (joint kinematics estimation) and analysis, the IMU-to-segment (I2S) assignment and alignment are central issues to obtain biomechanical joint angles. Existing approaches for I2S assignment usually rely on hand crafted features and shallow classification approaches (e.g., support vector machines), with no agreement regarding the most suitable features for the assignment task. Moreover, estimating the complete orientation alignment of an IMU relative to the segment it is attached to using a machine learning approach has not been shown in literature so far. This is likely due to the high amount of training data that have to be recorded to suitably represent possible IMU alignment variations. In this work, we propose online approaches for solving the assignment and alignment tasks for an arbitrary amount of IMUs with respect to a biomechanical lower body model using a deep learning architecture and windows of 128 gyroscope and accelerometer data samples. For this, we combine convolutional neural networks (CNNs) for local filter learning with long-short-term memory (LSTM) recurrent networks as well as generalized recurrent units (GRUs) for learning time dynamic features. The assignment task is casted as a classification problem, while the alignment task is casted as a regression problem. In this framework, we demonstrate the feasibility of augmenting a limited amount of real IMU training data with simulated alignment variations and IMU data for improving the recognition/estimation accuracies. With the proposed approaches and final models we achieved 98.57% average accuracy over all segments for the I2S assignment task (100% when excluding left/right switches) and an average median angle error over all segments and axes of 2.91° for the I2S alignment task.

## 1. Introduction

Inertial measurement units (IMUs) comprise accelerometers and gyroscopes providing measurements of 3D acceleration including acceleration due to gravity and 3D angular velocity. In most cases, they also contain magnetometers adding 3D magnetic fields. This is also referred to as MIMUs. Nowadays, IMUs are small in size and can be obtained at low cost. They are thus present in a multitude of devices. They also enjoy a widespread use in many different application areas, where humans wear IMUs on the body or nearby, e.g., inside the clothes. This includes, for instance, mobile health and biomechanics [1,2,3,4,5].

The following sections provide related work for the IMU-to-segment (I2S) assignment and alignment problems, first for human body motion capture, and then for activity recognition. Afterwards, related work is provided for applications with simulated and synthetic data. Finally, our scope and contributions are summarized.

### 1.1. I2S Assignment and Alignment for Inertial Body Motion Capture

**Figure 1:**
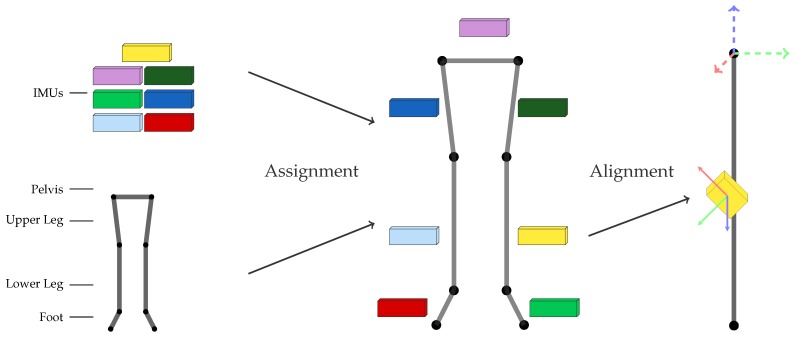
Illustration of the two addressed problems for seven lower body segments and seven IMUs: (i) I2S assignment; and (ii) I2S alignment determination.

In inertial body motion capture, the data from multiple IMUs, so-called IMU networks, are fused with a biomechanical model of (parts of) the human body to reconstruct segment orientations and joint angles [6,7] and sometimes also joint positions [8]. Figure 1 illustrates a kinematic lower body configuration. For obtaining biomechanical joint angles and positions, both the assignment of each IMU to the respective body segment as well as the position and orientation of each sensor relative to the assigned segment need to be known [7,9,10,11]. The latter is referred to as IMU-to-segment (I2S) alignment or I2S calibration (cf. Figure 1). A comparison of different sensor fusion methods for inertial body motion tracking (segment orientation estimation) regarding I2S alignment errors indicated that the position of the sensor relative to the segment is usually far less important for obtaining valid segment orientations (which are typically of more interest than the positions) than the I2S orientation [7]. Some methods were even shown to be independent of the IMU positioning in this study. In contrast, I2S orientation errors were shown to propagate at least linearly into the orientation estimation of the respective segment (and as a result into the derived joint angles), for all tested methods (without considering joint constraints). Therefore, we focus on I2S assignment and I2S orientation estimation (subsequently denoted I2S alignment) in this work.

Basically, all current I2S calibration procedures assume the I2S assignment to be known. Some approaches estimate an I2S assignment and consider an underlying body model in a hierarchical manner [12,13]. The first used decision trees on manually selected features with correlations of sensor data to subsequently assign 17 IMUs for a full body configuration or eight IMUs for a lower body or trunk configuration during walking trials. The accuracy of this method was between 97.5% and 100%, if the sensors were on the predefined positions in a suit. If sensors were missing or the positions were altered, the method either did not work or the accuracy decreased considerably (75.9% to 87.5%). Another recent approach assigned six IMUs to the lower limbs via constructed decisions based on walking characteristics and a proposed assignment hierarchy [13]. The accuracy of this approach ranged between 99.8% and 100% after three respectively five seconds of walking. However, the hierarchy needed multiple sensors. In contrast our approach considers each IMU individually and therefore scales to an arbitrary number of IMUs, including, for instance, only one IMU, seven IMUs (one on each considered lower body segment) or more than seven IMUs (multiple IMUs on one segment).

Assuming the I2S assignment to be known, obtaining a correct I2S calibration is still not trivial. Different calibration procedures have been proposed: static pose calibration that requires the user to take on specific poses [10], functional calibration [14,15], which requires the user to perform motions around predefined axes, or technical calibration that requires manual alignment of the IMUs with the bone structure [9]. In [9], typical I2S orientation calibration procedures were validated regarding their accuracy (trueness) and reproducibility (precision) relative to an optical reference system. The accuracy was in the range [8;26]° and precision was in the range [5;10]°. This attests the potentially large (human-induced) errors with respect to (w.r.t.) the I2S orientation, even when persons were instructed to perform the calibration procedure through experts. One recent approach aims at simultaneous joint kinematics and I2S calibration estimation, exploiting moving horizon optimization with additional constraints and priors for the calibration parameters [11].

### 1.2. I2S Assignment and Alignment for Activity Recognition, Deep Learning Approaches

Besides inertial body motion capture, another related application that is typically based on one or few IMUs, for instance from a mobile phone worn on the body or inside the cloths, is *human activity recognition* (HAR). Here, the aim is to infer activities, such as walking, sitting or running, from the sensor data. There are many classification approaches that use different manually derived features based on properties of the IMU signals, see, e.g., [16,17]. The classification accuracy largely depends on the feature selection which can be time consuming [18]. This process can also be automated together with the classification model, which leads to end-to-end learning. Here, the resulting model is directly applied to the raw sensor data. Deep learning approaches [19] are a prominent representative for end-to-end learning and have tremendous success in different applications fields, such as computer vision (e.g., image classification, action or gesture recognition [20,21,22,23,24]) or speech recognition [25,26]. There are also related applications to IMU based HAR [18,27], including the recently proposed deep learning framework for HAR. The latter is based on multimodal sensors such as IMUs. It combines local feature learning based on convolutional neural network (CNN) units with time sequence learning based on recurrent long-short-term memory (LSTM) units and is named *DeepConvLSTM* [18]. Moreover, CNN kernels were analyzed for automatic feature extraction with the aim to transfer these to different HAR domains in order to reduce training time significantly [28]. Our work is inspired by the DeepConvLSTM approach.

One problem for the majority of activity recognition approaches is that many expressive features for the same activity may differ largely, depending on the placement of the IMU relative to the body. For instance the IMU signals in a trouser pocket or at the pelvis are usually different from the signals at the feet in case of walking. This often renders HAR unreliable if the sensor placement is not known. One approach to face this problem is to use features that are robust to location and/or alignment. These are, however, usually less expressive and thus inferior for the recognition of complex motion [29]. In [29] sensor placement variations for wearable activity recognition were investigated in a structured way. The variations where subdivided into: (1) *on-body placement* (i.e., the place where the user carries the sensor on the body, this corresponds to our I2S assignment); (2) *within-body displacements* (i.e., the variation of the sensor placement on the respective segment); and (3) *orientation* (i.e., the device orientation w.r.t. the users body, this corresponds to our I2S orientation alignment). There are also approaches given to mitigate the errors introduced by the respective variations. Wrong on-body placement often renders an activity recognition approach unreliable. A study on optimal positions for HAR was performed in [30]. The within-body displacement can also decrease activity recognition accuracy. Remedy can be found, e.g., by dynamically choosing the most robust features [29,31] or by applying low-pass filtering after orientation correction [32]. Orientation variations yield unreliable rotation dependent features that, however, usually incorporate a lot more information to separate activity classes compared to rotation independent features such as the vector norm [29]. In [33] the effect of 16 IMU orientation variations and different IMU locations to an activity recognition approach was evaluated and severe accuracy decreases were found. A projection of the data to an approximate global frame as well as a model switch for different body locations was proposed for remedy. There are also approaches that classify the approximate location of one sensor [34,35,36,37] during walking and activities of daily living [38]. In [39] the sensor location and activity was jointly classified using sparse signal representation. Another method used accelerometer and gyroscope data to classify different on-body positions [40]. In [41] the orientation of an IMU w.r.t. the hip was classified considering rotations around one axis in 90° steps. The data were then transformed into the corresponding reference frame resulting in increased accuracy. Note, none of the mentioned approaches reconstructed the complete 3D orientation of the IMU relative to the respective body part, i.e., the I2S alignment. Performing this reconstruction has the advantage that the sensor data can always be rotated into the segment coordinate system, no matter how the sensor is oriented. For a data based approach this automatically introduces the challenge of having enough training data for the different possible orientations. The required amount of training data as well as class labels increases exponentially depending on the intended alignment classification accuracy. Therefore, our approach casts the I2S alignment task as a regression problem and makes use of artificial training data from simulated I2S alignment variations.

### 1.3. Applications with Simulated/Synthetic IMU Data

There are different frameworks for simulating IMU data from IMU trajectories [42,43], the latter, for instance, used recorded optical motion capture data. In the field of inertial body motion tracking, IMU data simulation is often used for evaluating different estimation methods (e.g., [7,8,44]).

In the field of computer vision, synthetic data were used to augment or create training data for increasing classification performance [45,46,47]. A typical challenge is the difference in feature distortions between synthetic and real data, i.e. the *synthetic gap* [45,47]. One way to face this problem is by using a multichannel autoencoder to learn the mapping between synthetic and real data [47]. A recent work [46] interpreted the learning of a neural network using synthetic data as learning of a proposal distribution generator for approximate inference in the synthetic-data generative model. This interpretation was used to explain model misspecifications that could be shown by only slight data variations, leading to a significant decrease in classification accuracy. The remedy was found by broadening the synthetic generator via adding an elastic displacement field to the synthetic data, which also lead to significant accuracy improvements [48].

Simulated IMU data, however, have not yet been used and investigated as database for deep learning approaches. In particular, the feasibility of transferring models learned on simulated IMU data to real data scenarios, has, to the best of our knowledge, not yet been investigated.

### 1.4. Contributions and Scope

Given the importance of sensor assignment and alignment determination in multiple areas, an automatic approach would, on the one hand, reduce the influence of human induced errors and, on the other hand, simplify the installation of an IMU system.

The contributions of this paper can be summarized as follows:

- We propose real-time capable deep learning approaches to solve the I2S assignment problem via classification and the I2S alignment problem via regression during walking for IMUs mounted on the lower body. The proposed approaches combine automatic feature learning via CNN units with time dynamic features based on two recurrent neural network approaches (LSTM and GRU). Since each IMU is handled individually, the proposed approaches scale to an arbitrary number of IMUs.
- We propose and evaluate methods for simulating IMU training data for a variety of I2S alignment orientations during walking using freely available [49] and newly recorded motion capture datasets. This is our approach for counteracting the problem of requiring the recording of exponentially many training data for sufficiently sampling potential I2S alignment variations.
- We investigate the performances of the proposed methods and approaches on simulated and real IMU test data, with different proportions of simulated and real IMU data used for training. The experiments show promising performances for both the classification and the regression problem with only a small amount of recorded IMU data (in terms of alignment variations).

In the following, the proposed methods, the datasets and experiments are introduced in Section 2 and Section 3, respectively. The results and a discussion are given in Section 4, and conclusions are drawn in Section 5.

## 2. Methods

First, the addressed problems are stated in Section 2.1. Then, the proposed network configurations with their different components are explained in Section 2.2. The involved coordinate frames and transformations are formalized in Section 2.3. Finally, Section 2.4 provides details on different aspects of our IMU data simulation.

### 2.1. Problem Statement

We address the following problems during *walking* motion (cf. Figure 1):

1. **I2S assignment**: Each IMU is separately assigned to the skeleton segment it is attached to. This results in the *classification problem* of mapping a window of (raw) IMU data to one of the seven classes associated to the lower body segments: (1) *LeftFoot*; (2) *LeftLowerLeg*; (3) *LeftUpperLeg*; (4) *Pelvis*; (5) *RightUpperLeg*; (6) *RightLowerLeg*; and (7) *RightFoot*.
2. **I2S alignment**: Each IMU’s orientation w.r.t. the associated segment is estimated via *regression* based on a window of IMU data. Note, I2S positions are assumed constant (on the middle of the respective segment) in this work.

In both cases, we use windows of 128 gyroscope and accelerometer data samples of the considered IMU as inputs. Moreover, throughout this work, the windows are always shifted by 16 data samples.

Note, successfully solving the stated problems provides I2S assignment and alignment of, in principle, any number of IMUs (they are treated separately) to the lower body skeleton in about two seconds of walking (assuming 60 Hz frequency) without using magnetometer data.

### 2.2. Proposed Networks

This section presents the network configurations proposed for solving the above stated problems. It starts with a general overview and then provides a mathematical formalization.

**Figure 2:**
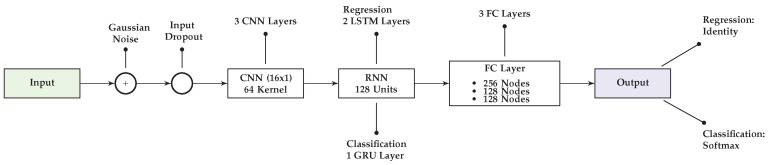
Overview of the proposed network configurations.

Figure 2 illustrates the structure of the proposed networks for both the classification and the regression task.

The configurations follow the general framework described in [18] with modifications described subsequently. *Input* denotes the input layer, i.e., (in this work) a window of 128 accelerometer and gyroscope data. *Gaussian Noise* refers to data augmenting noise samples, which we have introduced for processing simulated IMU data (cf. Section 2.4.1). A regularization based on *Input Dropout* has been introduced to avoid over-fitting (cf. Section 2.2.4). The main building blocks are the *CNN Layer* and the *RNN Layer*, where the latter is constructed differently for the classification and the regression problem. More details including the proposed modifications w.r.t. [18] are provided in Section 2.2.2 and Section 2.2.3. The respective final recurrent layer is densely connected with the output layer *Output* via a fully connected layer (*FC Layer*). The output layer either represents classes via probabilities using a *Softmax* layer (classification problem) or it maps to the components of a vector (regression problem), here representing the I2S alignment orientation. For the former, the class with the highest probability is chosen.

#### 2.2.1. Notation

A deep neural network with L>2 layers consists of an input layer with several input units ($a_{1:T}^{1}$), several hidden layers with activation units ($a_{1:T}^{l},1<l<$) and an output layer with units $a_{1:T}^{L}$. The latter might be a softmax layer for classification or an identity layer mapping to the previous layer for regression.

Given a sequence of *T* gyroscope ($x_{\mathrm{gyr}}\in R^{3}$) and accelerometer ($x_{\mathrm{acc}}\in R^{3}$) data of (without loss of generality) one IMU, we stack these data to obtain the *input data* $a_{1:T}^{1}=\left(a^{1}\left(1\right),\ldots ,a^{1}\left(T\right)\right)$, where $a^{1}\left(t\right)={\left(x_{\mathrm{acc}}{\left(t\right)}^{T},x_{\mathrm{gyr}}{\left(t\right)}^{T}\right)}^{T}$ denotes the IMU data at time *t*. We call the stacked components of the input units ($a_{j}^{1}\left(t\right)={\left(a^{1}\left(t\right)\right)}_{j},j=1,\ldots ,6$) *channels*.

A dense connection between the input layer (l=1) and the next layer at time *t* (i.e., a fully connected layer) can be formalized as

(1) $$\begin{array}{c} a^{2}\left(t\right)=\sigma \left(W_{a,a}^{1}a^{1}\left(t\right)+b^{1}\left(t\right)\right). \end{array}$$

Here, $a^{2}\left(t\right)$ are the activations of layer 2, and $W_{a,a}^{1}$ is the weight matrix of layer 1 with the subscript denoting a connection from one activation to another activation. Moreover, ${\left(W_{a,a}^{1}\right)}_{i,j}$ represents the weight between activation *j* in layer 1 and *i* in layer 2. Further, $a^{1}\left(t\right)$ are the channels of the input data, $b^{1}\left(t\right)$ are the biases and σ is the non-linear activation function, which is typically applied to each component of the resulting vector separately. The specific functions are given in the subsections below.

#### 2.2.2. Convolutional Neural Network (CNN) Layer

A CNN layer convolves (or often cross-correlates) activations of a layer, e.g., the input layer, with kernels (filters). CNNs with multiple layers perform convolutions on the respective activations of the previous layer. The convolution with the kernel yields a weight sharing between activations, which has different advantages for data with neighborhood relations (either spatially, temporally or both, depending on the kernel used). First, the amount of parameters to be estimated is reduced compared to a fully connected layer. Second, through the weight sharing, the generalization of the “learned” convolution kernel and the resulting *feature map*, i.e., the activation map at the next layer, is usually increased (if enough data is available). CNNs are thus often employed to learn features automatically. They can adapt well to locally disturbing effects like noises and slight biases, given a large enough kernel size.

The following formalization is inspired by [18]. To simplify notation, we consider the convolution over one channel of the input sequence. The convolution is then applied for each channel separately. A CNN with a single layer extracts features from the input signal through a convolution operation of the signal with a kernel. In a CNN, the activation of a unit represents the result of the convolution of the kernel with the complete input signal of the considered window (or activation of the previous layer in the case of stacked CNNs). The kernels are optimized as part of the supervised training process. The dimensionality of the kernel depends on the input data and their neighborhood relations. We use 1D kernels in this work as previously proposed for temporal convolution [4]. We consider a feature map as an array of units that share the same parameterization (weight vector and bias). We denote the amount of feature maps per layer *l* as $F^{l}$. At the input layer, we have $F^{1}=1$ for each channel of the input signal, resulting in one feature map. Further feature maps are introduced in layers l≥2 to be able to learn different filters/kernels to respond to different patterns. In what follows, the term $K_{k,f}^{l}$ denotes a 1D kernel convolved over feature map *f* in layer *l* to create feature map *k*. With the activation of feature map *f* at unit τ in layer *l* ($a_{f}^{l}\left(\tau \right)$), this convolution can be written as

(2) $$\begin{array}{c} {\left(K_{k}★a\right)}_{f}^{l}\left(\tau \right)=\sum_{p=-P/2}^{P/2}a_{f}^{l}\left(\tau -p\right)K_{k,f}^{l}\left(p\right). \end{array}$$

Here, *P* is the width of the kernel. The feature map *k* of layer l+1 at unit τ can be obtained from the feature maps of the previous layer *l*, including the above defined convolution, as follows

(3) $$a_{k}^{\left(l+1\right)}\left(\tau \right)=\sigma \left(BN\left(\sum_{f=1}^{F^{l}}{\left(K_{k}★a\right)}_{f}^{l}\left(\tau \right)+b_{k}^{l}\left(\tau \right)\right)\right)\;.$$

Here, σ(a)=max(a,0) is a rectified linear unit (ReLu) activation function, BN denotes the batch normalization per activation as further detailed in [50]—it normalizes the convolution output by mean and variance—and $b_{k}^{l}\left(\tau \right)$ represents the bias.

Note, a model with several convolutional layers in a stacked configuration (where the output of layer *l* is the input for layer l+1) may be able to learn a hierarchical representation of the data, where deeper layers represent the inputs in a more and more abstract way [18].

**Figure 3:**
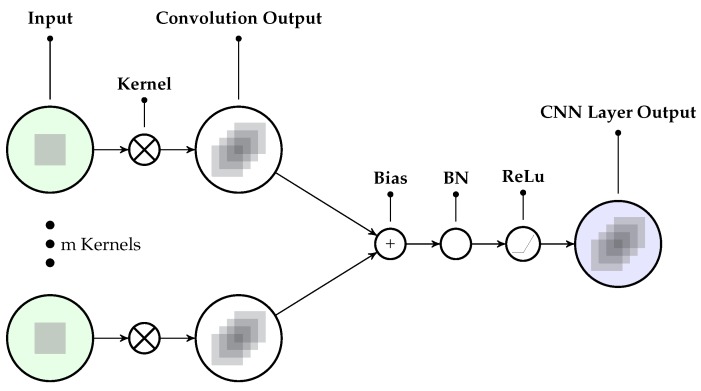
The CNN layer configuration used in the proposed networks. Note, the Bias, batch normalization (BN) and rectified linear unit (ReLu) operations are applied per activation (see Equation (3)).

Compared to [18], besides a slightly larger kernel size (cf. Table A1 for all hyper parameters), the main difference of the proposed CNN layers is the inclusion of the normalization (BN) to push the activations closer to zero mean and unit variance. In our tests, this gave better results compared to the original convolution layers used in [18]. An explanation for this might be the differences in the data characteristics when mixing simulated and real IMU data for training. Note, network configuration tests are further detailed in Section 4.1. Figure 3 illustrates the convolution from the input to the second layer.

#### 2.2.3. Recurrent Neural Network (RNN) Layer

The (feed forward) CNN layers presented in Section 2.2.2 are well suited for learning locally robust features in a time sequence. To represent and learn temporal dynamics of arbitrarily long sequences, a so called RNN has shown to be efficient both regarding its representation power and the required amount of parameters and input data [19,25]. Recurrent neural networks consist of cells with recurrent connections, i.e., forming a directed circle. A drawback of this approach is the problem of vanishing and exploding gradients for long-term dependencies [51]. While there are different approaches to prevent this, we use LSTM and GRU layers, as proposed by [25,52]. In our evaluation the LSTM approach showed the most beneficial behavior for the considered regression problem, while the GRU approach performed best for the classification problem, with comparably less parameters. Note, in [18], only LSTMs were used. Both approaches are further detailed and formalized in Appendix A.

#### 2.2.4. Dropout Layer

To regularize the proposed model, we tested different dropout (statistical regularization) techniques [53]. It was shown that dropout is, on the one hand, more computational efficient than training multiple models for model averaging. On the other hand, it was shown to perform very well in combination with other regularizations, such as $L_{2}$-Regularization and Early Stopping [54]. Thus, we evaluated the impact of $L_{2}$-Regularization and Early Stopping as regularization methods in addition to dropout, on the same dataset as used for the other configurations, described in Section 4.1. Regarding dropout we tested input dropout with a keep probability of 0.8 [54], naive dropout between the fully connected layers and variational dropout between the RNN layers (latter both with keep probability of 0.5) [55], by taking the same dropout mask in each time step on the input, output and recurrent connections. We achieved the best results with an input dropout of 0.8 and no dropout between RNN cells as well as between the fully connected layers, in combination with an L2 Regularization with a small weight (see Table A1 in Appendix A).

#### 2.2.5. Application of the Proposed Networks

Applying the above described network configurations to the considered problems has the following implications. For the I2S assignment problem, the output layer is a linear activation with seven hidden nodes that enter the softmax layer resulting in seven probabilities. Here, the highest probability is chosen to represent the predicted IMU location on the lower body (i.e., the I2S assignment). As objective function, we used a cross entropy loss function, during training. Solving the I2S alignment problem requires a suitable rotation parametrization. In order to circumvent the inclusion of additional constraints, we compared two minimal parameterizations in a plausibility test: axis angle and stereographic projection of a unit quaternion [56]. While the former representation is more widespread, it has a singularity around the identity rotation, which can lead to numerical instabilities, if it is not specifically handled. Moreover, the derivatives include irrational sine and cosine terms that are nonlinear and need some sort of approximation during optimization, which can deteriorate convergence and accuracy [56]. The stereographic projection of a quaternion provides a minimal parameterization, for which the derivatives are rational and more. Moreover, there is no singularity for the identity rotation. This parametrization also gave significantly better results in our plausibility tests, which convinced us to use this representation for all further tests. Since the stereographic projection of a unit quaternion is a 3D vector, the output layer mentioned in Section 2.2 is a linear activation with three hidden nodes, the output of which is the predicted I2S alignment. As objective function, we used an L2 loss function, during training.

### 2.3. Coordinate frames and transformations

**Figure 4:**
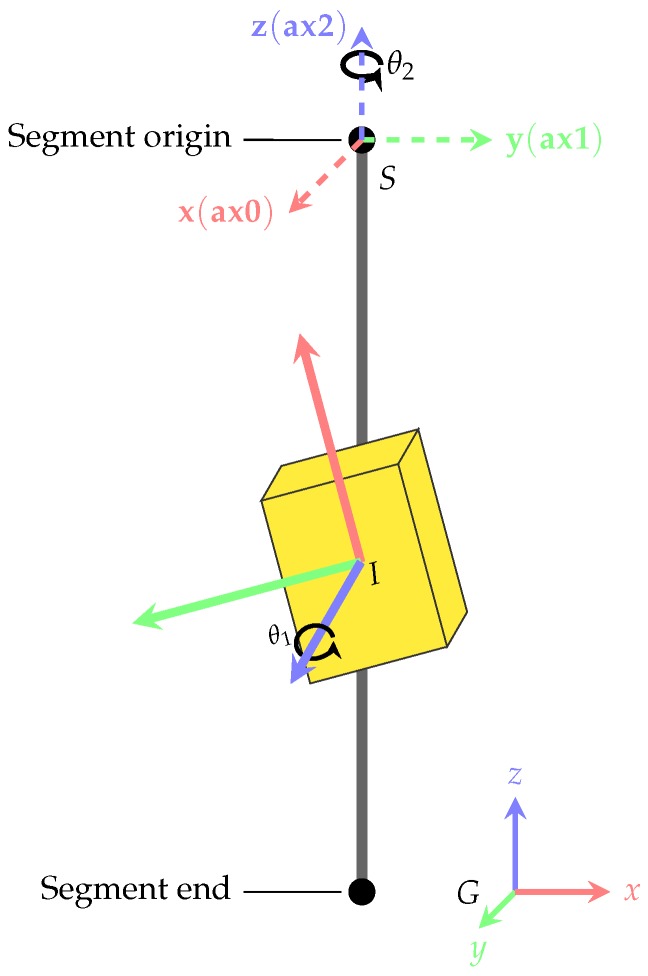
Exemplary illustration of IMU (*I*), segment (*S*, dashed) and global (*G*) coordinate frames for one IMU and one segment. The I2S alignment variations in terms of axes and angles ($\theta_{1}$, $\theta_{2}$) as basis for IMU data simulation are also illustrated.

We use three different coordinate frames: The global frame *G* is a fixed reference frame, *S* refers to the coordinate frame attached to the considered skeleton segment, and *I* refers to the coordinate frame attached to the considered IMU (cf. Figure 4). Moreover, the quantities $R^{GS},S^{G}$ and $R^{GI},I^{G}$ denote the poses (orientations and positions) of the considered segment and IMU coordinate frames w.r.t. the global frame. From these, the respective I2S orientation (alignment) and position can be obtained as

(4) $$\begin{array}{cc} R^{SI} & =R^{SG}R^{GI}={\left(R^{GS}\right)}^{T}R^{GI} \end{array}$$

(5) $$\begin{array}{cc} I^{S} & =R^{SG}\left(I^{G}-S^{G}\right). \end{array}$$

In addition, given 3D kinematics data in terms of global segment poses ($R^{GS},S^{G}$) and I2S poses ($R^{SI},I^{S}$), the IMU poses can be obtained as

(6) $$\begin{array}{cc} R^{GI} & =R^{GS}R^{SI} \end{array}$$

(7) $$\begin{array}{cc} I^{G} & =R^{GS}I^{S}+S^{G}. \end{array}$$

These relations are required for IMU data simulation as detailed in the following Section 2.4. Moreover, $R^{SI}$ denotes the I2S alignment for a given IMU that we want to predict.

### 2.4. Creation of Artificial Training Data

A major part of this work concerns the mixing of simulated and real IMU training data for increasing the accuracy of the proposed networks w.r.t. the I2S assignment and alignment tasks without the need to record large amounts of training data with alignment variations. This section details the creation of artificial training data in terms of simulated IMU data from available 3D kinematics data (global segment orientations and positions). Here, lower body kinematics data for walking was obtained through own recordings with a commercial IMU based system [57] as well as from an available motion capture dataset [49] (see Section 3.1 for further details on the used datasets). For both sources, the kinematics data were mapped to the skeleton shown in Figure 1. For creating artificial training data, the first step consisted of simulating a suitable set of I2S alignments for each body segment (Section 2.4.1). Note, in this work, we considered variations of the I2S orientations ($R^{SI}$), while keeping the I2S positions ($I^{S}$) stationary on the midpoints of the respective segment axes (i.e., inside the bones). The second step consisted of using these I2S alignments together with the global segment poses available from the 3D kinematics data to obtain sets of IMU trajectories for each segment, from which ideally simulated IMU data was obtained through differentiation. This was followed by mimicking different measurement artifacts to obtain realistic IMU data. The latter steps are detailed in Section 2.4.2.

#### 2.4.1. Simulation of I2S Alignment Variations

An illustration of the I2S alignment variation simulation for one segment is shown in Figure 4. The basic idea is to, in a structured way, generate orientation variation samples for each IMU and associated segment according to the assumptions that body segments can be approximated by capsule like surfaces and IMUs are aligned tangentially to these surfaces, i.e., that their *z*-axes are approximately aligned with the outward pointing surface normals [11]. This can be achieved by systematically sampling rotation angle tuples ${\left(\theta_{1},\theta_{2}\right)}_{l}\in R\times R,l=1,\ldots ,N$ with R being a set of $\sqrt{N}$ equidistant angle samples (of the full circle) and applying these to both the *z*-axis of the considered IMU (in the IMU coordinate frame) and the axis that connects segment origin and end, represented in the respective segment coordinate system. Formalizing this results in alignment variations ${\hat{R}}_{l}^{SI},\;l=1,\ldots ,N$ with

(8) $$\begin{array}{c} {\hat{R}}_{l}^{SI}=R_{a}\left(\theta_{2,l}\right)R^{SI}R_{z}\left(\theta_{1,l}\right),l=1,\ldots ,N. \end{array}$$

Here, $R^{SI}$ is the assumed initial I2S alignment, e.g., an actual alignment used during an IMU based recording and obtained from an N-pose calibration [10]. The symbol $R_{x}\left(\theta \right)$ denotes a rotation of angle θ around rotation axis x and *N* depends on the angle step size. For the experiments, we used $R=\{0^{\circ },45^{\circ },90^{\circ },\ldots ,315^{\circ }\}$ leading to N=64 alignment variations. Note, while $R_{z}$ always corresponds to the IMU’s *z*-axis (represented in the IMU coordinate frame), $R_{a}$ depends on the body location and is chosen as a=z (i.e., the segment’s *z*-axis represented in the segment coordinate system) for left and right upper and lower legs, a=y for the pelvis and $a={\left(0.9,0.0,-0.4\right)}^{T}$ for the feet. The latter approximates the vector between the foot’s origin and the head of the second metatarsal bone projected onto the ground [8]. These choices correspond to the assumed skeleton definition (segment coordinate systems), as further detailed in Section 3.1.

#### 2.4.2. Simulation of Realistic IMU Data

Given a time sequence of global segment poses (i.e., 3D kinematics data) and a set of constant I2S poses as obtained from the procedure described in the previous section (i.e., neglecting soft tissue artifacts), time sequences of IMU poses were calculated using Equation (6). From this, ideal IMU datasets were simulated using standard data differentiation [58]. Note, in order to compensate for measurement artifacts in the used motion capture data, the global segment pose data was preprocessed with a zero lag Butterworth filter of order 8 and a cutoff frequency of 10 Hz [59]. Moreover, quantization artifacts (sensor ranges and digital resolution) were mimicked according to the specifications of the actual IMUs used in this work [57].

**Figure 5:**
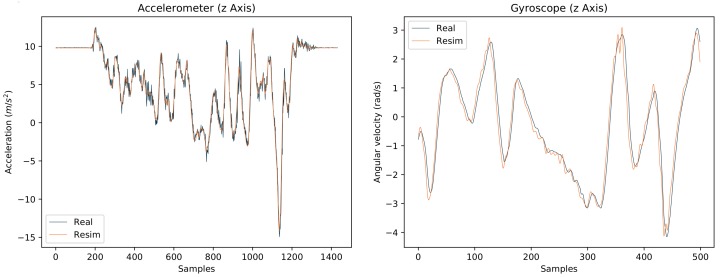
Comparison between real and re-simulated IMU data for one example from a capturing setup where IMUs were rigidly mounted on a rigid body and were additionally tracked with a marker based optical system. The frequency was 60 Hz, i.e., 60 samples correspond to one second.

Figure 5 illustrates an example of good correspondence between real and re-simulated IMU data for a plausibility test, where IMUs were rigidly mounted on a rigid body (i.e., excluding soft tissue artifacts) moved freely in space and were additionally tracked with a gold standard marker based optical system [60]. The Pearson correlation coefficients between real and re-simulated accelerometer and gyroscope data were on average for all channels and IMUs 0.99 and 0.97, respectively (see Table A2 and Table A3 in Appendix B).

We also compared our results to the simulated IMU data obtained from IMUSim [42] with comparable results (Pearson correlation coefficients when using [42]: 0.91 and 0.97, respectively).

**Figure 6:**
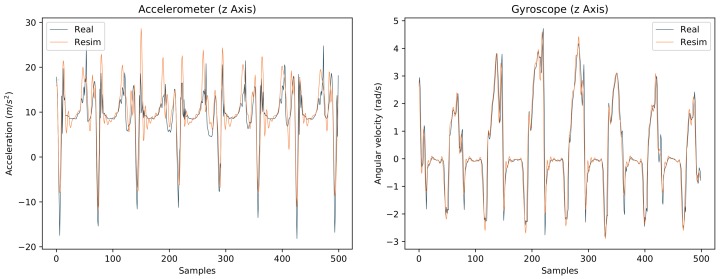
Comparison between real and re-simulated IMU data from the right foot from a capturing setup where IMUs were mounted on a person during walking and were additionally tracked with a marker based optical system. The frequency was 60 Hz, i.e., 60 samples correspond to one second.

Figure 6 shows an example comparison of real and re-simulated data from a walking motion (IMU on the right foot). Here, the average Pearson correlation coefficients over all channels and all IMUs were 0.57 for the accelerometers and 0.93 for the gyroscopes (see Table A4 and Table A5 in Appendix B). The comparably lower correlation coefficient for the accelerometers is shown in Figure 6. Here, the greatest differences between real and re-simulated data are observed during the ground impacts.

**Figure 7:**
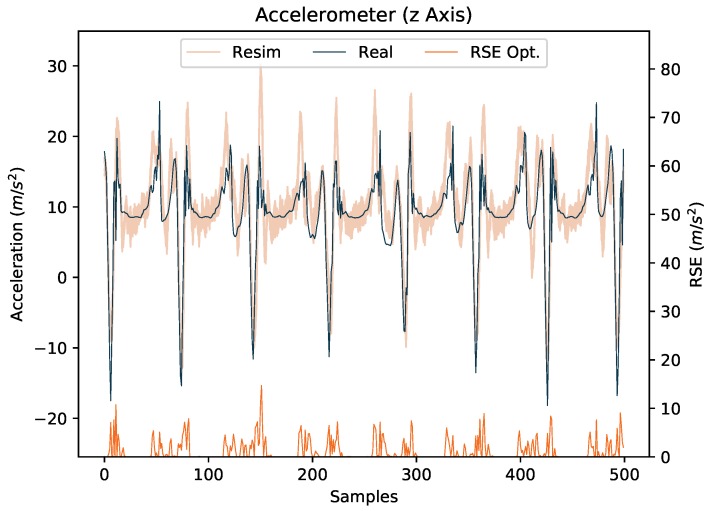
Figure 6 augmented with 100 noisy re-simulated signals per timestep and the respective root squared errors (RSE) between the real signal and the closest re-simulated signal at each timestep (RSE Opt.).

This synthetic gap can be a result of additional artifacts due to clothing or soft-tissue (causing additional accelerations), which are most severe during ground impacts. The approximated I2S position assumption can also add artifacts. To initially address this, inspired by [61], we added zero-mean Gaussian noise ($e_{a}∼N\left(0,\sigma^{2}\right)$, with $\sigma^{2}$ = 1 m/s${}^{2}$) to all accelerometer channels. Note, we considered the gyroscope data to be reasonable without additional noise, in this work. Figure 7 augments Figure 6 by showing 100 re-simulated noisy signals per timestep. In addition, the absolute errors between real and re-simulated signals are shown.

Qualitatively speaking, it can be seen in the figure that the real accelerometer signal is often included in the fan of noisy re-simulated signals. Quantitatively, this shows in a drop of the average RMSE over all channels and all IMUs from 4.02 m/s${}^{2}$ for a signal without noise to 1.23 m/s${}^{2}$ when considering the closest noise sample at each timestep (i.e., the optimal case). Anticipating the experimental results in Section 4, the addition of Gaussian noise resulted in a considerable improvement w.r.t. the addressed I2S assignment and alignment tasks (8 percent and 1.5 degrees, respectively, for purely training networks on (noisy) simulated IMU data and testing on unseen labeled real IMU data). Hence, we kept this setting for the subsequent experiments and left more sophisticated soft tissue models (e.g., [61]), general motion artifact compensation (e.g., [62]) or specific compensation of walking artifacts to future work.

## 3. Datasets and Experiments

This section introduces the datasets used to train and evaluate the proposed networks (Section 3.1), the training configuration and time (Section 3.2) as well as the error metrics used for evaluation (Section 3.3).

### 3.1. Datasets

**Figure 8:**
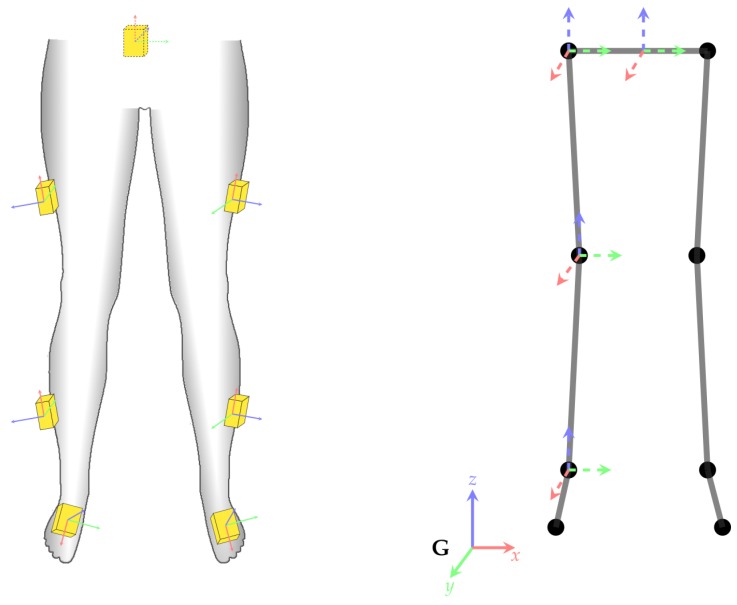
(**Left**) IMU configurations used for the recording of dataset **C** (dashed lines mark IMUs placed on the back); and (**Right**) skeleton with exemplary segment coordinate systems of pelvis and right leg (analogous for left leg). The global coordinate system (*G*) is a fixed reference coordinate system.

Three datasets (**A**,**B**, and **C**) were used for training and evaluation. Dataset **A** extracts walking motions from the publicly available CMU dataset [49]. The latter provides 3D kinematics data captured with a marker based optical motion capture system. The other datasets consist of IMU and 3D kinematics data recorded with the Xsens Awinda MVN Studio software and the Xsens Awinda IMU hardware [57]. More details are given in the following:

**A**  IMU data simulated from the 3D kinematics data of parts of the CMU dataset (42 participants performing different walking styles, see Table A6 in Appendix C). Note, this dataset is distinguished by a high amount of simulated IMU configuration variations (as described in Section 2.4) based on an already high amount of variability in the 3D kinematics data (in terms of persons and walking styles) used for simulation.
**B**  Collected real IMU data from four male participants (mean age: 32, mean weight: 82 kg, mean height: 181 cm) instructed to walk for one minute back and forth (within an area of five by five meters), with nine different IMU configurations as detailed in Appendix C (Table A7).
**C**  Collected real IMU data from 28 participants (13/15 male/female, mean age: 25 years, mean weight: 70 kg, mean height: 176 cm) instructed to walk for six minutes in an eight-shape within an area of about five by five meters, with one standard IMU configuration, different from the previously mentioned nine configurations (see Figure 8). This study was approved by the Ethical Committee of the University of Kaiserslautern and written informed consent was obtained from all participants prior to their participation.

### 3.2. Training

In this work, we utilized methods from the Tensorflow library [63] for training and evaluation of the proposed network configurations. The training was performed on Nvidia GPUs with an average training time of twelve hours for the I2S assignment model and 48 hours for the I2S alignment model. Further details regarding the choice of the optimizer and different hyper parameters can be found in Appendix D (Table A1).

### 3.3. Evaluation

For model evaluation disjoint *training*, *validation* and *test* sets were created from the above mentioned datasets. According to the leave-one-subject-out (LOSO) method, the test sets consisted of persons not used for training. The training and validation sets were created with a 95% to 5% splitting. The training data were used to train the model. Evaluation on the same data allows verifying whether the model is able to reconstruct the labels of the training data. The validation data were used to evaluate the model on unseen data during the training process. Finally, the test data were used to evaluate the model on data from a person that was not present during the training process. Together, this provides indications of the model’s ability to generalize to unseen data and users.

Two different sets of error measures were used for evaluating the classification and the regression approach. The performance of the classification model was assessed via three scalar measurements based on the classification output in comparison to the ground truth dataset labels, i.e.,

(9) $$\mathrm{precision}=\frac{\mathrm{true}\;\mathrm{positives}}{\mathrm{true}\;\mathrm{positives}+\mathrm{false}\;\mathrm{positives}}\;,$$

(10) $$\mathrm{recall}=\frac{\mathrm{true}\;\mathrm{positives}}{\mathrm{false}\;\mathrm{positives}+\mathrm{false}\;\mathrm{negatives}}\;,$$

and

(11) $$f_{1}=\frac{\mathrm{precision}\cdot \mathrm{recall}}{\mathrm{precision}+\mathrm{recall}}\;.$$

The performance of the regression model was assessed by comparing the predicted I2S alignment orientation quaternions (${\tilde{q}}_{i}$) with the ground truth quaternions ($q_{i}$) for each window *i*. The orientation deviations ($q_{i}\cdot {\tilde{q}}_{i}^{-1}$) were decomposed into (absolute) angular deviations ($\left|\alpha_{err,i,ax0}\right|$, $\left|\alpha_{err,i,ax1}\right|$, $\left|\alpha_{err,i,ax2}\right|$) around the *x*-, *y*-, *z*-coordinate axes of the respective segment coordinate frame using an Euler angle decomposition of order XYZ (cf. Figure 4). From these measures (which are in the range $\left[0^{\circ },180^{\circ }\right]$) we calculated typical statistics including minimum, maximum, mean, median, and mean squared angular errors in the considered dataset. Note, we also use box plots to illustrate the errors. Here, a white circle denotes the mean, a line across the box denotes the median, a colored bounding box denotes the upper (>75%) and lower (<25%) quantiles, and whiskers of 1.5 times the inter quantile range denote the upper and lower bounds.

## 4. Results and Discussion

This section details the results of applying the proposed methods to the different datasets while moving from simulated to real IMU data. Note, the hyper parameters summarized in Appendix A (Table A1) were obtained through cross validations on dataset **A** and were used for all experiments. Section 4.1 first provides an evaluation of different network configurations on dataset **A**, i.e., simulated IMU data for training and evaluation. Section 4.2 then provides evaluation results for the models trained with the proposed (best performing) network configurations (as illustrated in Figure 2) on dataset **A**. These are subsequently denoted *pre-trained models*. Thereafter, Section 4.3 evaluates the effects of combining the pre-trained models with simulated and real IMU data for training when testing on real IMU data. This uses dataset **B**. Finally, Section 4.4 provides an evaluation of the final models using all datasets.

### 4.1. Evaluation of Network Configurations on Dataset ***A***

**Figure 9:**
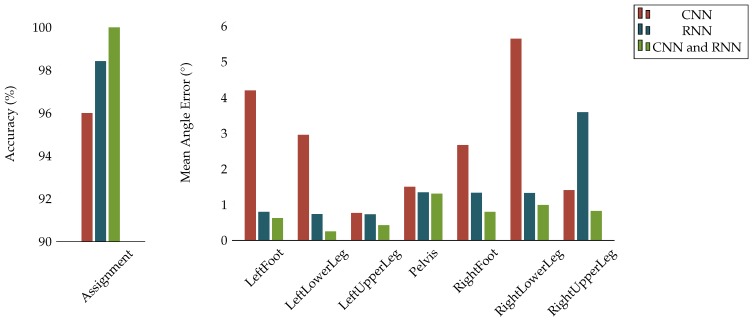
Network configuration cross validation on dataset **A**: (Left) The bar plot represents the accuracy of the I2S assignment problem; and (Right) the bar plots represent the mean angle errors over all axes and windows for the I2S alignment problem for each body segment.

The network configurations described in Section 2.2 (and motivated by [18]) combine CNN and RNN layers. This aims for a model that allows to extract local IMU signal patterns via the CNN layers as well as long-term dependencies via the RNN layers. To evaluate the performances of different network configurations in our considered problems, we performed cross validations using only CNN layers, only RNN layers and a combination of both based on dataset **A**. The results for both the I2S assignment and alignment tasks are provided in Figure 9 and in Appendix D (Table A8 and Table A9). They clearly confirm the superiority of the proposed combination also for our problems.

### 4.2. Pre-trained Models Based on Dataset ***A***

**Figure 10:**
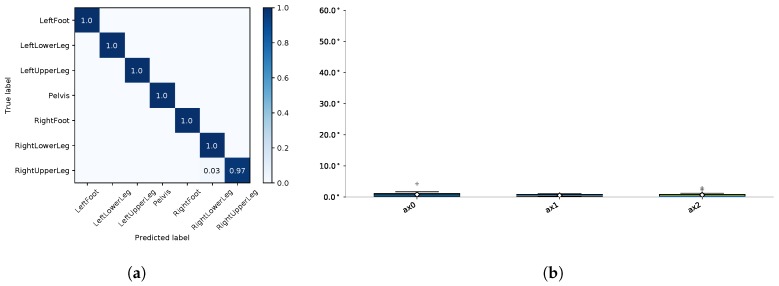
Evaluation results on simulated dataset **A**: (**a**) confusion matrix for the I2S assignment problem; and (**b**) boxplots of the angle errors around the three body segment axes for the I2S alignment problem (exemplary for the left foot).

The next step consisted of model training and evaluation on dataset **A**, i.e., training and testing on simulated IMU data. Figure 10a shows the results for the I2S assignment problem in terms of a confusion matrix. The model achieved 99.99% precision, recall and f-score. Figure 10b shows the results for the I2S alignment problem in terms of box plots of the absolute angular deviations (angle errors) around the three body segment coordinate axes exemplary for the left foot. The mean error over all windows and axes was 0.62° and the maximum error was 4.23° (cf. Figure 9 (right) for average results concerning the other segments).

These results attest a good performance of the trained models under controlled conditions (simulated IMU data). Note that we already included Gaussian noise to cover additional real-world effects (cf. Section 2.4.1).

The models trained on dataset **A** correspond to a proposal distribution generator for an approximate inference from a synthetic data generative model [46], which created the motion samples of dataset **A**. In the following, these models are denoted *pre-trained models*.

### 4.3. Impact of Mixing Real and Simulated IMU Training Data Based on Dataset ***B***

To investigate the effects of combining simulated and real IMU data for training when testing on real IMU data from a person not seen during training, we performed four tests based on dataset **B**:

1. Training on real IMU data from three persons (nine alignment variations each, see Table A7 in Appendix C), testing on real IMU data from the left out (test) person (nine alignment variations).
2. Training on re-simulated IMU data from three persons (64 simulated alignment variations per real IMU alignment), testing on real IMU data from the test person (nine alignment variations).
3. Setup 2, but with additionally using the real IMU data captured from the three participants (nine alignment variations) for training (i.e., 1/65 of the training data is based on real IMU data).
4. Setup 3, with the training being warm-started with the pre-trained models of Section 4.2.

Here, test case 1 can be considered as baseline (training and testing on only few real IMU data), while cases 2 through 4 represent different strategies of using simulated IMU data for increasing the I2S alignment variations present in the training dataset.

**Figure 11:**
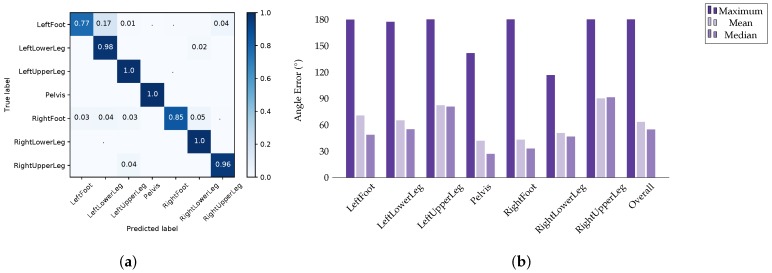
Evaluation results on dataset **B**, test case 1: (**a**) confusion matrix for the I2S assignment problem; and (**b**) angle errors over all windows, axes and considered (nine) IMU configurations for the I2S alignment problem for test case 1.

Figure 11a shows the results of test case 1 for the I2S assignment problem. The average accuracy was 94% with the main source of inaccuracy being the feet. Figure 11b shows the results for the I2S alignment problem in terms of per segment mean, median and maximum angle errors over all windows, axes and considered IMU configurations. Here, the model performed rather poorly, with an overall (over all segments) mean angle error of 63.33°, average median angle error of 54.62° and maximum angle error of 179.99°. This indicates that the training data did not contain sufficient alignment variations for the proposed regression approach.

The following sections describe the effects of augmenting the real IMU training data with simulated training data when testing on real IMU data from an unseen person (test cases 2 through 4), first for the I2S assignment then for the I2S alignment problem.

#### 4.3.1. I2S Assignment Problem

**Figure 12:**
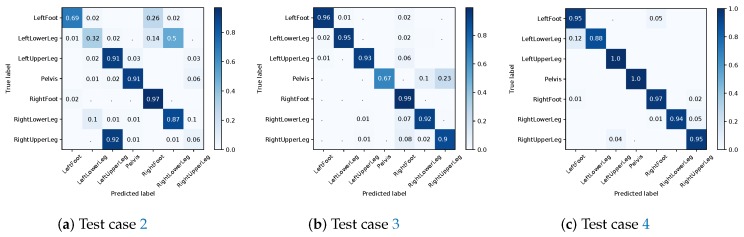
Evaluation results in terms of confusion matrices for the I2S assignment problem on dataset **B**.

Training on purely simulated data from few participants and then testing on real IMU data from an unseen participant (test case 2) gave an overall accuracy of 68% for the I2S assignment problem. This is a significant decrease compared to the baseline test case 1. Note, the majority of mis-classifications are due to left/right switches that are not explicitly handled in this approach (see Figure 12a). Hence, the prior distribution induced via the few simulated IMU data seems to be insufficient for a high classification accuracy. Adding a small amount of real IMU data to the training set (test case 3) resulted in a performance increase to an average accuracy of 92% (see Figure 12b). Note, the accuracy is still slightly lower compared to the baseline test case 1 (real training data only). Using the pre-trained model from Section 4.2 (based on dataset **A**) resulted in an average accuracy of 96% (see Figure 12c). This last model performed best with an additional accuracy improvement of 4% as compared to test case 3 (without pre-training) and an increase of 2% compared to the baseline test case 1.

In summary, these results indicate that a pre-training with simulated IMU data from an existing motion capture dataset (dataset **A**) in combination with a mixture of simulated and real IMU training data from a small additionally captured dataset (dataset **B**) yield already promising results for the I2S assignment problem in a real data scenario.

#### 4.3.2. I2S Alignment Problem

**Figure 13:**
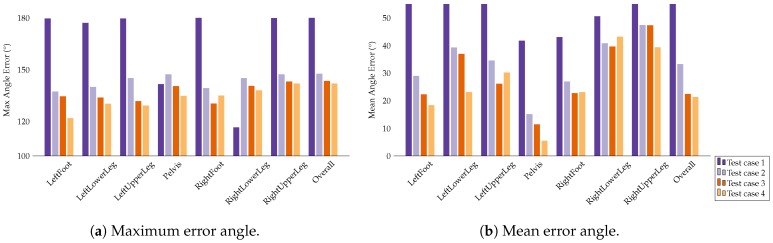

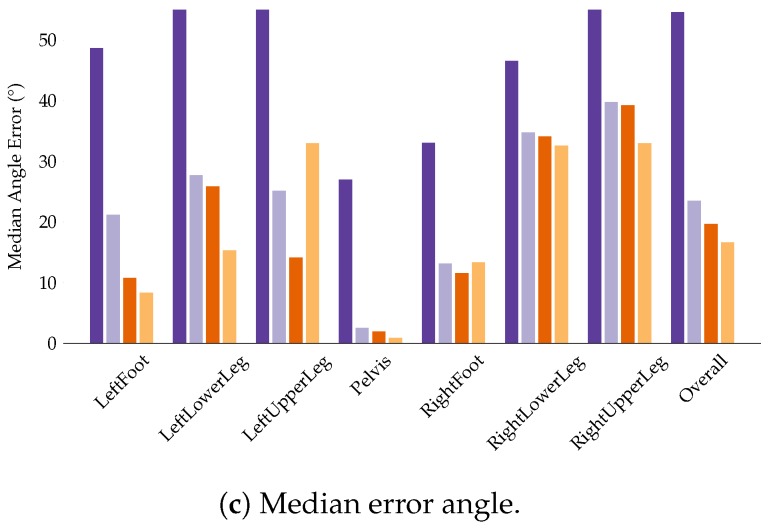
Evaluation results for the I2S alignment problem on dataset **B**. The bar plots show the maximum, mean and median angle errors for all segments over all windows, axes and considered (nine) IMU configurations. These were obtained through cross validation.

Figure 13 summarizes the evaluation results for test cases 1 through 4 for the I2S alignment problem. Training on purely simulated IMU data (test case 2) resulted in an overall (over all segments) mean angle error over all windows and axes of 33.30°. The maximum was 147.64° and the average median was 23.46°. This already constitutes a considerable improvement as compared to the baseline test case 1. Adding real IMU data to the training set (test case 3) resulted in a further reduction of the angle errors. The overall mean angle error reduced to 22.5°, the maximum to 143.13° and the average median to 18.11°. Finally, a slight additional improvement was achieved by using the pre-trained model from Section 4.2 (test case 4). Here, the overall mean, maximum and average median angle error further reduced to 21.35°, 142.03° and 16.59°, respectively. In summary, these results indicate that our proposed approaches of simulating IMU data and combining the result with real IMU data for training can considerably increase the I2S alignment estimation accuracy when using only a small specifically captured dataset (dataset **B**).

### 4.4. Evaluation of the Final Models

In a last step, final models were trained across all datasets. More specifically, test case 4 in Section 4.3 was extended with real and simulated IMU training data based on dataset **C**. The final models were tested with two test persons, one randomly selected from dataset **B** and one randomly selected from dataset **C**, both not used for training. Hence, the test data included ten different IMU configurations. The following sections describe the evaluation results, first for the I2S assignment then for the I2S alignment problem.

#### 4.4.1. I2S Assignment Problem

**Figure 14:**
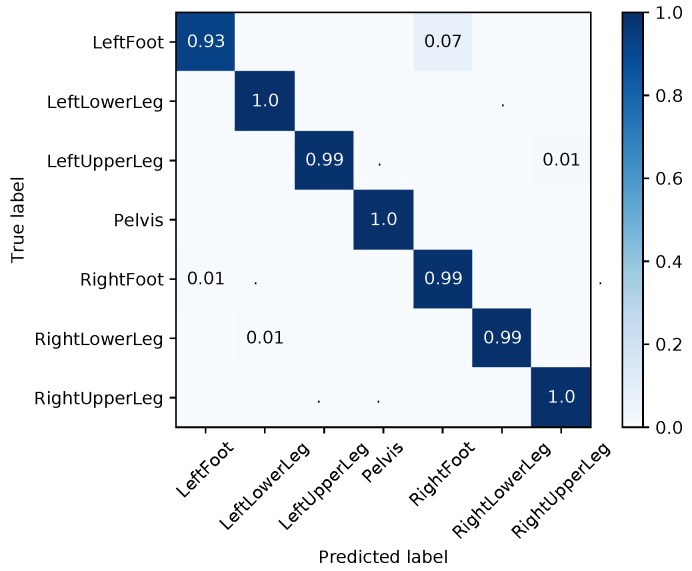
Evaluation results in terms of a confusion matrix for the I2S assignment problem using the final model (based on all datasets).

**Figure A2:**
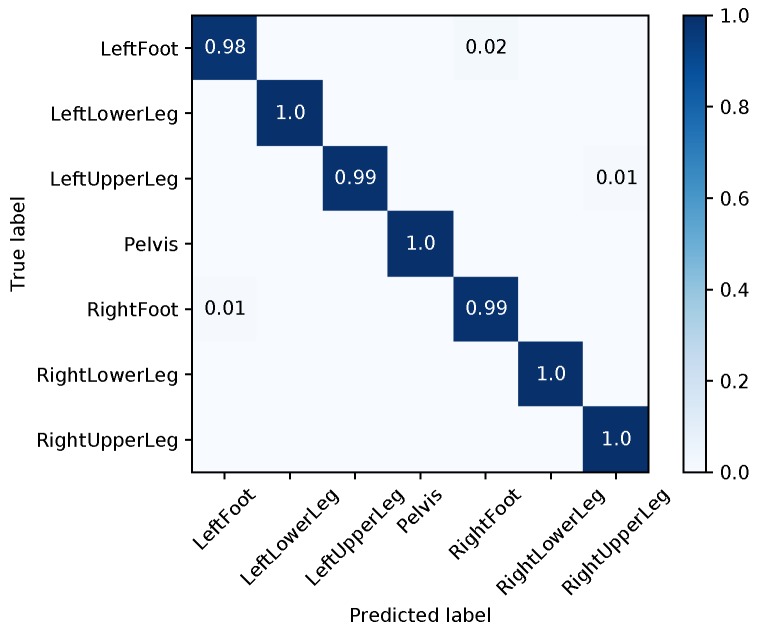
I2S assignment problem, final model: test person from dataset **B**, IMU configuration 1 (cf. Table A7). Average accuracy: 99.57%.

**Figure A3:**
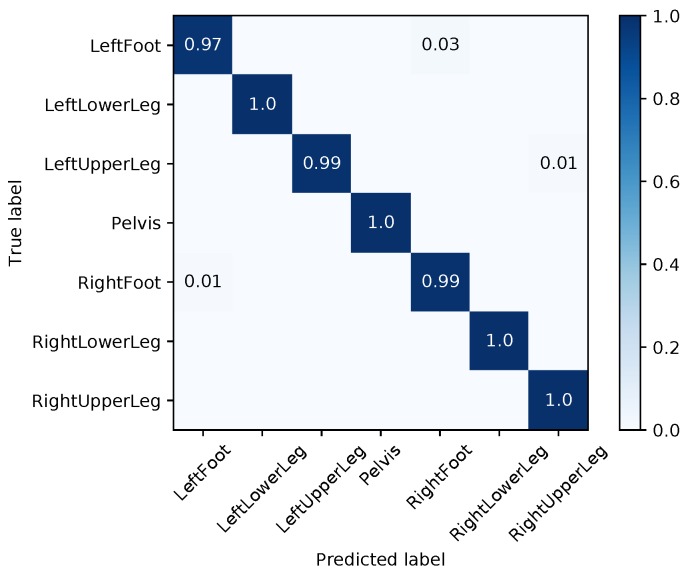
I2S assignment problem, final model: test person from dataset **B**, IMU configuration 2 (cf. Table A7). Average accuracy: 99.43%.

**Figure A4:**
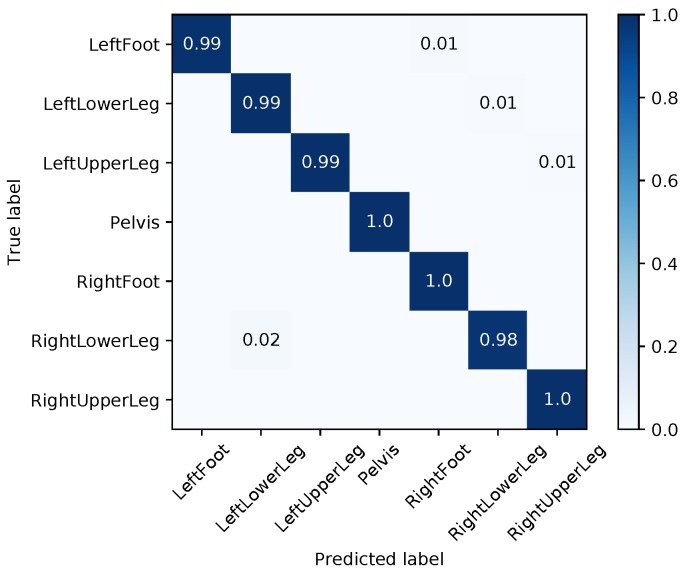
I2S assignment problem, final model: test person from dataset **B**, IMU configuration 3 (cf. Table A7). Average accuracy: 99.43%.

**Figure A5:**
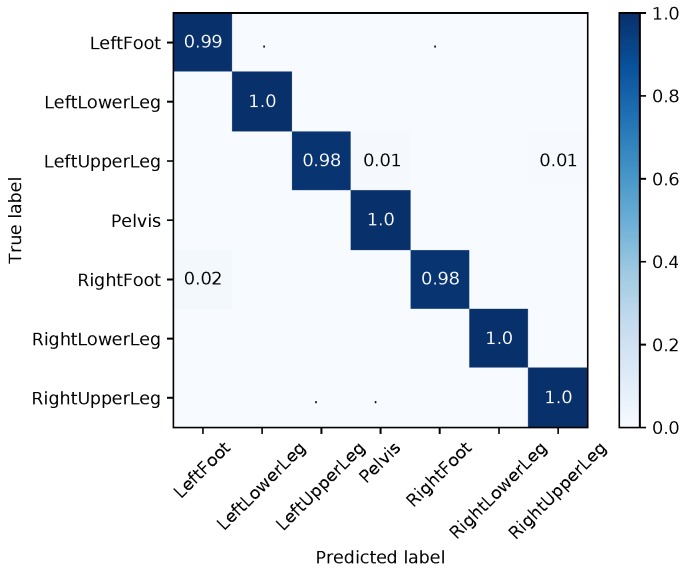
I2S assignment problem, final model: test person from dataset **B**, IMU configuration 4 (cf. Table A7). Average accuracy: 99.43%.

**Figure A6:**
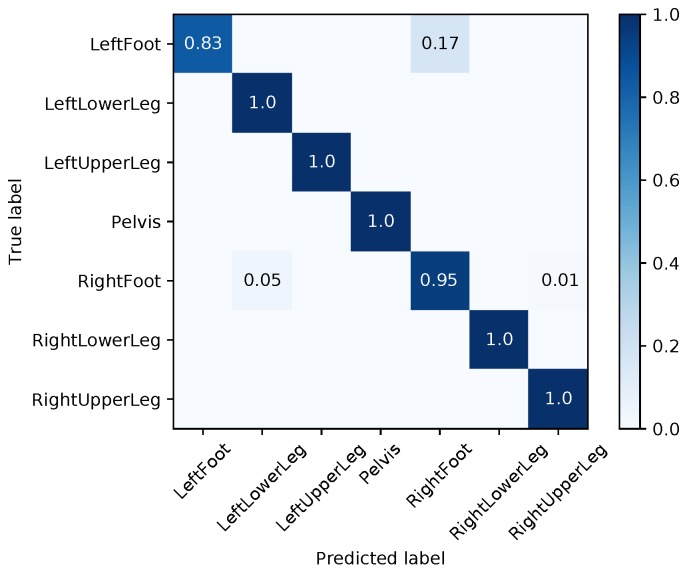
I2S assignment problem, final model: test person from dataset **B**, IMU configuration 5 (cf. Table A7). Average accuracy: 97.29%.

**Figure A7:**
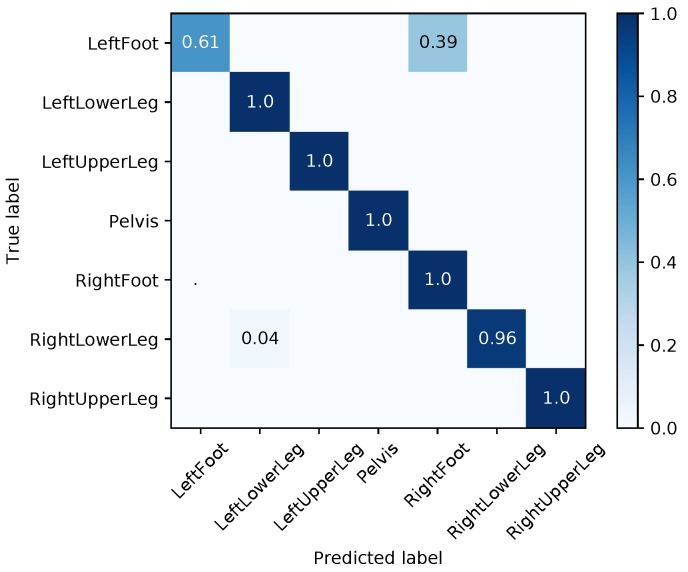
I2S assignment problem, final model: test person from dataset **B**, IMU configuration 6 (cf. Table A7). Average accuracy: 96.14%.

**Figure A8:**
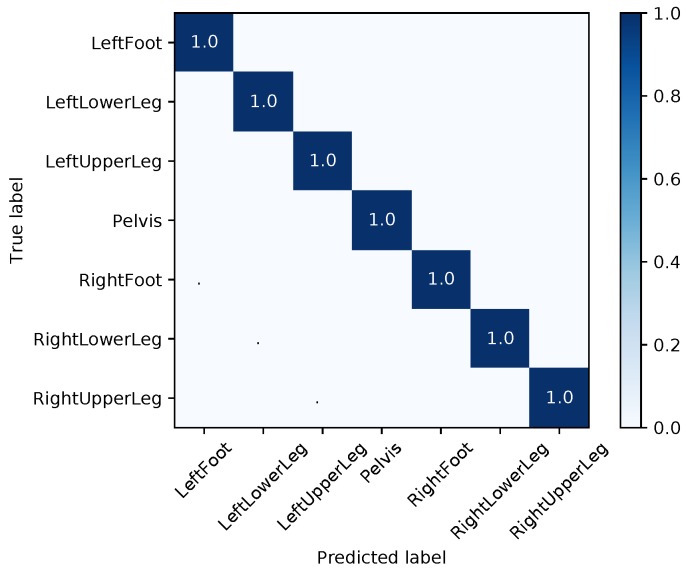
I2S assignment problem, final model: test person from dataset **B**, IMU configuration 7 (cf. Table A7). Average accuracy: 100%.

**Figure A9:**
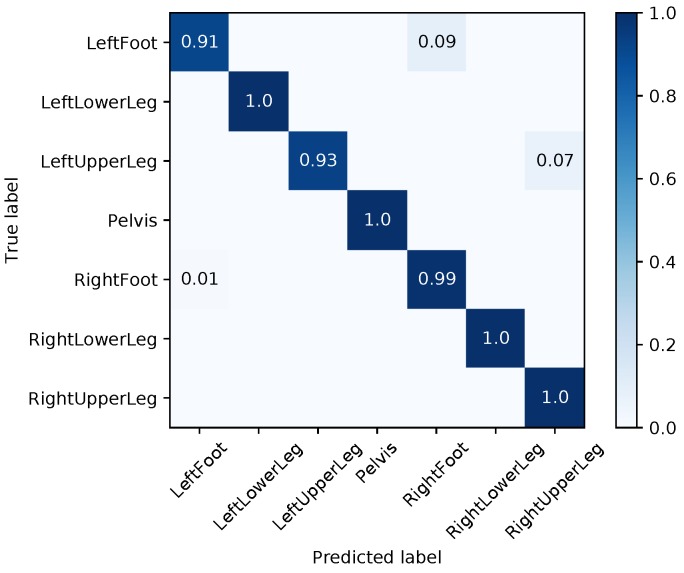
I2S assignment problem, final model: test person from dataset **B**, IMU configuration 8 (cf. Table A7). Average accuracy: 98.14%.

**Figure A10:**
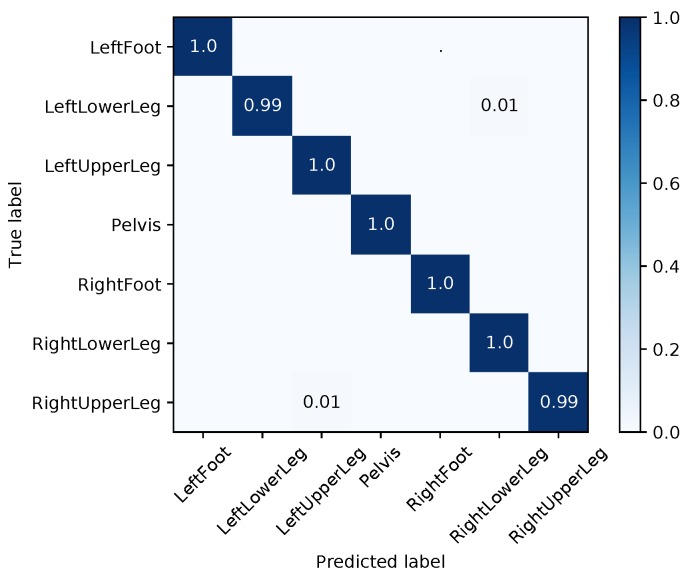
I2S assignment problem, final model: test person from dataset **B**, IMU configuration 9 (cf. Table A7). Average accuracy: 99.71%.

**Figure A11:**
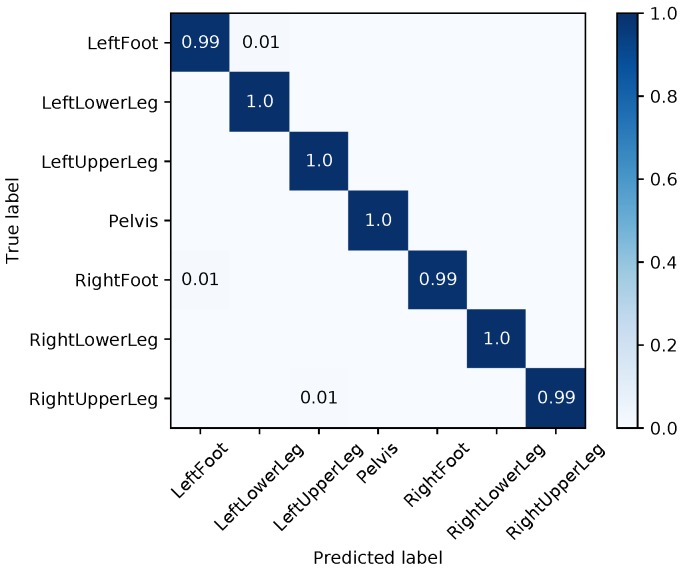
I2S assignment problem, final model: test person from dataset **C**, IMU configuration according to Figure 8). Average accuracy: 99.14%.

Figure 14 shows the evaluation results (over all test persons/IMU configurations) for the I2S assignment problem. The average accuracy was 98.57%. We also performed separate evaluations for each different sensor configuration in the test data. The results in terms of confusion matrices are all given in Appendix E (Figure A2, Figure A3, Figure A4, Figure A5, Figure A6, Figure A7, Figure A8, Figure A9, Figure A10 and Figure A11). The average accuracies over all IMUs ranged between 96.14% and 100%. These results attest a consistently high accuracy for the I2S assignment for a variety of IMU assignments and alignments with our proposed approach. Note that errors *only* occurred due to left/right leg switches, which are hard to distinguish or even indistinguishable for some cases (if the segments are treated individually as done in our approach).

#### 4.4.2. I2S Alignment Problem

**Figure 15:**
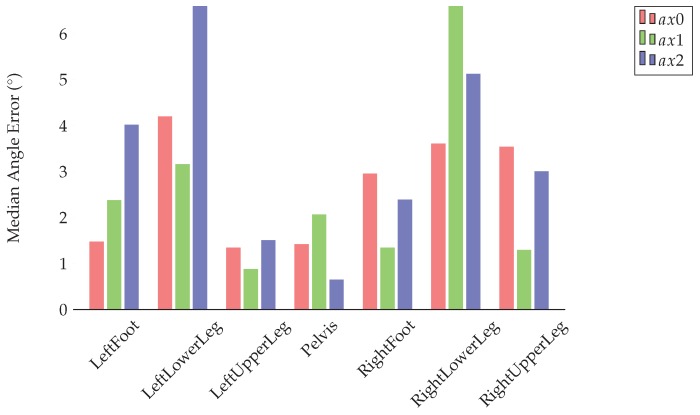
Evaluation results for the I2S alignment problem using the final model (based on all datasets). The bar plots show the median angle errors around the three body segment axes over all windows, considered test persons and IMU configurations.

Figure 15 summarizes the evaluation results for the I2S alignment problem in terms of the average median angle errors for all axes over all windows, test persons and IMU configurations. With an overall (over all windows, axes, segments, test persons and IMU configurations) mean angle error of 15.21° and average median of 2.91°, the results further improved considerably compared to test case 4 in Section 4.3.2 (the maximum was slightly higher with 168.58°) (see Table A10 in Appendix E for additional results). Hence, the addition of a higher amount of real IMU data (with one IMU configuration) completed with simulated alignment variations further increased the degree of generalization of the trained networks.

## 5. Conclusions

The aim of this work was to investigate the potential of deep learning for the challenging tasks of I2S assignment and alignment w.r.t. a biomechanical lower body model based on windows of IMU data (angular velocities, accelerations). We used only small data windows (128 samples) in order to obtain a real-time capable approach. Moreover, we considered each IMU separately in order to obtain an easily adaptable approach for applications in both inertial body motion capture (which is often based on multiple IMUs) and human activity recognition (which often uses a single IMU). Moreover, we aimed at using only a limited amount of real IMU data (in terms of I2S alignments) for training in order to obtain a practical approach.

We confronted these challenges by combining real IMU data samples with additional artificially generated IMU data based on available and newly captured 3D kinematics data. The generation of artificial IMU data was based on the simulation of I2S alignment variations followed by the simulation of noisy IMU data, where the noise was added to reduce the synthetic gap. For both the I2S assignment and alignment determination we utilized a suitable combination of CNN and RNN (LSTM and GRU) based neural network approach.

Regarding the evaluation results on real IMU data, the most promising approach consisted of combining simulated and real IMU data for training, while warm-starting the training with models pre-trained on a purely simulated dataset.

For the I2S assignment problem we achieved 94% average accuracy on a small newly recorded dataset with only four persons and nine I2S alignment variations. This was improved to 96% by augmenting the real training data with simulated I2S alignment variations and by starting from a pre-trained model based on purely simulated IMU data (see Section 4.3). With a further increase of the amount of real IMU data (eight-shaped walking from 28 persons and one standard I2S alignment) we obtained an average accuracy of 98.57% (see Section 4.4). Note, the remaining errors were only due to left/right switches. The segments (pelvis, upper leg, lower leg, and foot) where identified with 100% accuracy. Hence, a reliable left/right assignment is one part of our future work.

For the I2S alignment problem we achieved significant improvements when mixing real and simulated IMU data for training. When training and testing on the newly captured dataset with only four persons and nine I2S alignment variations the angle errors were rather large, e.g., 63.33° overall mean angle error, 179.99° overall maximum angle error and 54.62° overall median angle error (Section 4.3). This, however, considerably improved to an overall mean of 21.35°, maximum of 142.03° and median of 16.59° when augmenting the real training data with simulated I2S alignment variations and by starting from the pre-trained model (see Section 4.3). When increasing the amount of real IMU data for training as mentioned above the proposed approach showed an additional improvement (overall mean and median angle errors of 15.21° and 2.91°, respectively). The observed outliers (overall maximum of 168.58°) were likely caused by real-world effects that were not yet sufficiently covered by the training dataset (e.g., amount of real I2S alignment variations and movement variations, soft tissue artifacts, and I2S position offsets).

Altogether, these results demonstrate the feasibility of augmenting real IMU training data with simulated data for improving the recognition/estimation accuracies. Note that the proposed approach provides I2S assignment and alignment of, in principle, any number of IMUs (they are treated separately) to the lower body skeleton in about two seconds of walking (assuming 60 Hz frequency) without using magnetometer data.

To further improve the validity and reliability of the proposed approach, there are a few interesting avenues for future research: the inclusion of uncertainties into the proposed approach, e.g., based on Bayesian deep learning [53] to provide indications for reliable I2S alignment estimates; a reliable left/right assignment; and an extension to the full body. In general, a more sophisticated approach for IMU data simulation, e.g., based on more realistic body shapes [64], a better soft tissue model, possibly such as the one used in [61], and the modeling of I2S position offsets could further reduce the synthetic gap in IMU data simulation and by this the amount of outliers and the required amount of real data. 

## Figures and Tables

**Figure A1:**
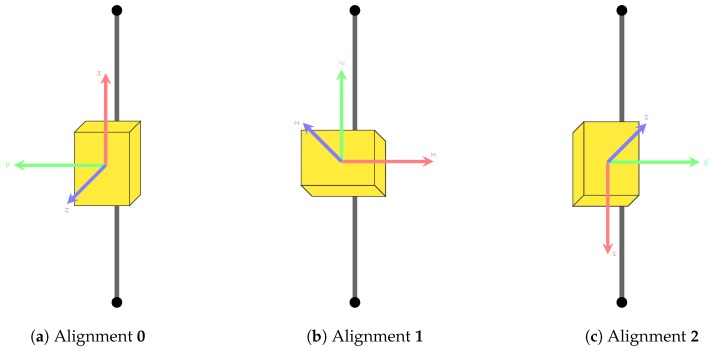
IMU alignments used during the recording of dataset **B**.

## Appendix A. Proposed Network Configurations

### Appendix A.1. Long Short-Term Memory (LSTM)

The core of the LSTM networks are the LSTM cells that replace the recurrent connections in the RNN approach with gated cells. The idea is that the recurrence is jointly trained with gates, which in turn control the information flow through the cell and are therefore able to counteract the vanishing and exploding gradient problem. LSTMs have been shown to learn long time dependencies more easily than RNNs. Note that these dependencies can also reach over a considered input window of data. The input to the LSTM cell consists of an activation at time *t* (a(t)) and the output of the previous hidden state of the cell h(t−1). The hidden state is usually initialized with h(0)=0. The LSTM cell consists of different components. The *cell state* c(t) is updated in a controlled manner, via different gates. The *forget gate* controls the amount of information from past cell states that should be prevented from influencing future cell states. It is updated as

(A1) $$\begin{array}{c} f\left(t\right)=\sigma_{f}\left(W_{a,f}a\left(t\right)+W_{h,f}h\left(t-1\right)+b_{f}\right). \end{array}$$

Here and in the following, the subscripts of the matrices denote the from-to relationship [18], i.e., $W_{a,f}$ is a matrix that relates the (input) activation vector to the vector **f**(t) representing the forget gate. $W_{h,f}$ relates the hidden state h(t−1) and **f**(t). The activation function $\sigma_{f}$ is a tangens-hyperbolicus (tanh) function and $b_{f}$ denotes a bias. The *input gate*i(t) controls the amount of information that is allowed to modify the cell state via the hidden state and current activation. It is updated as

(A2) $$\begin{array}{c} i\left(t\right)=\sigma_{i}\left(W_{a,i}a\left(t\right)+W_{h,i}h\left(t-1\right)+b_{i}\right). \end{array}$$

As above, $W_{a,i}$ and $W_{h,i}$ are matrices, $b_{i}$ is a bias vector and $\sigma_{i}$ a tanh function. The cell state is updated with a conditional self-loop, weighted by the forget gate vector and the update weighted by the input gate vector

(A3) $$\begin{array}{c} c\left(t\right)=f\left(t\right)⊙c\left(t-1\right)+i\left(t\right)⊙\sigma_{c}\left(W_{a,c}a\left(t\right)+W_{h,c}h\left(t-1\right)+b_{c}\right). \end{array}$$

Here, ⊙ denotes an element-wise multiplication. Again, $W_{a,c}$ and $W_{h,c}$ denote matrices, $b_{c}$ denotes a bias vector and $\sigma_{c}$ is a tanh function. Finally, the *output gate* vector and the hidden state at the next timestep (h(t)) are computed as

(A4) $$\begin{array}{ccc} o(t) & = & \sigma_{o}\left(W_{a,o}a+W_{h,o}h\left(t-1\right)+W_{c,o}c\left(t\right)+b_{o}\right), \end{array}$$

(A5) $$\begin{array}{ccc} h(t) & = & o\left(t\right)⊙\sigma_{h}\left(c\left(t\right)\right). \end{array}$$

Again, $W_{h,o}$ and $W_{c,o}$ are matrices, $b_{o}$ is a bias vector and $\sigma_{o},\sigma_{h}$ are tanh functions.

### Appendix A.2. Gated Recurrent Unit (GRU)

In contrast to an LSTM cell, the GRU cell, as introduced in [52], fuses forget and input gate to an *update gate*. Further, it merges the cell and hidden states. This results in a reduced amount of parameters and, hence, a simpler model. Similar to the LSTM cell, a new state is computed as a linear sum between the previous and current determined state, although GRU cells do not provide a mechanism for controlling the degree of state exposure. The state, h(t), is computed as

(A6) $$\begin{array}{c} h\left(t\right)=\left(1-z\left(t\right)\right)⊙h\left(t-1\right)+z\left(t\right)⊙\tilde{h}\left(t\right), \end{array}$$

where $\tilde{h}\left(t\right)$ denotes the *candidate state* and z(t) the update gate. The update gate, which determines the degree to which the previous cell state and the candidate state contribute to the cell update, is updated as

(A7) $$\begin{array}{c} z\left(t\right)=\sigma_{z}\left(W_{a,z}a\left(t\right)+W_{h,z}h\left(t-1\right)\right), \end{array}$$

where $W_{a,z}$ and $W_{h,z}$ are weight matrices, as in Appendix A.1. The candidate state $\tilde{h}\left(t\right)$ is related to the calculation of a traditional recurrent unit as in [65]. It is updated as

(A8) $$\begin{array}{c} \tilde{h}\left(t\right)=\sigma_{\tilde{h}}\left(W_{a,\tilde{h}}a\left(t\right)+r\left(t\right)⊙\left(W_{r,\tilde{h}}h\left(t-1\right)\right)\right). \end{array}$$

Here, r(t) represents the *reset gate*, $W_{a,\tilde{h}}$ and $W_{r,\tilde{h}}$ are matrices, $\sigma_{\tilde{h}}$ is a tanh function and a(t) denotes the activation. Similar to the update gate z(t), the reset gate is computed as

(A9) $$\begin{array}{c} r\left(t\right)=\sigma_{r}\left(W_{a,r}a\left(t\right)+W_{h,r}h\left(t-1\right)\right), \end{array}$$

where $W_{a,r}$, $W_{h,r}$ are matrices and $\sigma_{r}$ is a tanh function. This finishes the update of the GRU cell.

### Appendix A.3. Hyper Parameters

**Table A1:** Hyper parameters of the proposed network configurations, determined through cross validation based on dataset **A** and used for all tests.

		Assignment	Alignment
Optimizer	Batch size	256	256
	Learning rate	0.001	0.001
	Optimizer	Adam	Adam
CNN Layer	# Layer	3	3
	Kernel	16 × 1	16 × 1
	# Kernel	64	64
RNN Layer	# Layer	1	2
	Cell	GRU	LSTM
	# Units	128	128
Regularization	$L_{2}$-Factor	0.0001	0.0001
	Keep probability	0.8	0.8

## Appendix B. IMU Data Simulation

**Table A2:** Pearson correlation coefficients and root mean squared errors (RMSE) between real and re-simulated accelerometer data from a capturing setup where the seven IMUs used in the experiments were rigidly mounted on a rigid body and were additionally tracked with a marker based optical system.

**Accelerometer (Pearson Correlation Coefficient)**				
**IMU**	x	y	z	**mean**
1	0.9903	0.9915	0.9862	0.9893
2	0.9901	0.9898	0.9819	0.9873
3	0.9913	0.9909	0.9854	0.9892
4	0.9918	0.9828	0.9762	0.9836
5	0.9917	0.9875	0.9819	0.9870
6	0.9897	0.9869	0.9756	0.9841
7	0.9908	0.9920	0.9866	0.9898
**mean**	**0.9908**	**0.9888**	**0.9820**	**0.9872**
**Accelerometer RMSE [m/s${}^{2}$]**				
	x	y	z	**mean**
1	0.6950	0.6969	0.7934	0.7285
2	0.7190	0.7977	0.9613	0.8260
3	0.6506	0.6991	0.7894	0.7130
4	0.6814	0.9577	1.0750	0.9047
5	0.6640	0.8111	0.9024	0.7925
6	0.7534	0.9265	1.2443	0.9747
7	0.6869	0.6644	0.7574	0.7029
**mean**	**0.6929**	**0.7933**	**0.9319**	**0.8060**

**Table A3:** Pearson correlation coefficients and root mean squared errors (RMSE) between real and re-simulated gyroscope data from a capturing setup where the seven IMUs used in the experiments were rigidly mounted on a rigid body and were additionally tracked with a marker based optical system.

**Gyroscope (Pearson Correlation Coefficient**)				
**IMU**	x	y	z	**mean**
1	0.9594	0.9760	0.9778	0.9711
2	0.9637	0.9793	0.9737	0.9722
3	0.9645	0.9800	0.9759	0.9734
4	0.9617	0.9798	0.9753	0.9723
5	0.9640	0.9798	0.9750	0.9729
6	0.9632	0.9816	0.9776	0.9742
7	0.9652	0.9800	0.9766	0.9739
**mean**	**0.9631**	**0.9795**	**0.9760**	**0.9729**
**Gyroscope RMSE [rad/s]**				
**IMU**	x	y	z	**mean**
1	0.5978	0.3519	0.2661	0.4053
2	0.5671	0.3252	0.2904	0.3942
3	0.5604	0.3210	0.2782	0.3865
4	0.5840	0.3236	0.2811	0.3962
5	0.5636	0.3231	0.2834	0.3901
6	0.5838	0.3060	0.2700	0.3866
7	0.5558	0.3193	0.2745	0.3832
**mean**	**0.5732**	**0.3243**	**0.2777**	**0.3917**

**Table A4:** Pearson correlation coefficients and root mean squared errors (RMSE) between real and re-simulated accelerometer data from a capturing setup where IMUs were mounted on a person during walking and were additionally tracked with a marker based optical system.

**Accelerometer (Pearson Correlation Coefficient)**				
**IMU**	x	y	z	**mean**
LeftLowerLeg	0.7428	0.4830	0.6028	0.6095
RightUpperLeg	0.5726	0.2803	0.7139	0.5223
LeftUpperLeg	0.6717	0.1602	0.7737	0.5352
RightLowerLeg	0.7325	0.3326	0.5148	0.5266
LeftFoot	0.8067	0.3962	0.6565	0.6198
Pelvis	0.8132	0.2409	0.4940	0.5161
RightFoot	0.8456	0.4342	0.8026	0.6942
**mean**	**0.7407**	**0.3325**	**0.6512**	**0.5748**
**Accelerometer RMSE [m/s${}^{2}$]**				
**IMU**	x	y	z	**mean**
LeftLowerLeg	3.6462	4.3371	4.1111	4.0314
RightUpperLeg	4.2053	3.2582	3.8274	3.7637
LeftUpperLeg	3.1212	3.9498	3.4068	3.4926
RightLowerLeg	3.9041	5.0482	4.3344	4.4289
LeftFoot	5.3138	4.9329	4.7109	4.9859
Pelvis	2.2677	2.5007	2.3478	2.3720
RightFoot	4.6748	6.4197	4.0363	5.0436
**mean**	**3.8762**	**4.3495**	**3.8250**	**4.0169**

**Table A5:** Pearson correlation coefficients and root mean squared errors (RMSE) between real and re-simulated gyroscope data from a capturing setup where IMUs were mounted on a person during walking and were additionally tracked with a marker based optical system.

**Gyroscope (Pearson Correlation Coefficient)**				
**IMU**	x	y	z	**mean**
LeftLowerLeg	0.8720	0.9802	0.9628	0.9383
RightUpperLeg	0.8736	0.9570	0.9207	0.9171
LeftUpperLeg	0.8661	0.9692	0.8901	0.9085
RightLowerLeg	0.8681	0.9820	0.9469	0.9323
LeftFoot	0.9145	0.9618	0.9234	0.9332
Pelvis	0.9778	0.8890	0.9349	0.9339
RightFoot	0.8451	0.9673	0.9504	0.9209
**mean**	**0.8882**	**0.9581**	**0.9327**	**0.9263**
**Gyroscope RMSE [rad/s]**				
**IMU**	x	y	z	**mean**
LeftLowerLeg	0.8678	0.5459	0.2285	0.5474
RightUpperLeg	0.6663	0.4652	0.1804	0.4373
LeftUpperLeg	0.8404	0.3579	0.2251	0.4745
RightLowerLeg	0.7338	0.5556	0.1653	0.4849
LeftFoot	0.6386	1.0709	0.4129	0.7075
Pelvis	0.1287	0.1257	0.0938	0.1161
RightFoot	0.4638	1.0177	0.4836	0.6550
**mean**	**0.6199**	**0.5913**	**0.2557**	**0.4890**

## Appendix C. Datasets

**Table A6:** List of the 42 participants and recordings used from the CMU dataset [49] to create dataset **A**.

Participant ID	Recording	Frames	Duration [s]	Motion Description
02	01	313	313.	Walk
	02	298	298.	Walk
03	01	432	432.	Walk on uneven terrain
	02	365	365.	Walk on uneven terrain
	03	4563	4563.	Walk on uneven terrain
	04	4722	4722.	Walk on uneven terrain
05	01	598	598.	Walk
06	01	494	494.	Walk
07	01	316	316.	Walk
	02	329	329.	Walk
	03	415	415.	Walk
	04	449	449.	Slow walk
	05	517	517.	Slow walk
	06	417	417.	Walk
	07	379	379.	Walk
	08	362	362.	Walk
	09	306	306.	Walk
	10	301	301.	Walk
	11	315	315.	Walk
08	01	277	277.	Walk
	02	309	309.	Walk
	03	353	353.	Walk
	04	484	484.	Slow walk
	06	296	296.	Walk
	08	283	283.	Walk
	09	293	293.	Walk
	10	275	275.	Walk
10	04	549	549.	Walk
12	01	523	523.	Walk
	02	673	673.	Walk
	03	565	565.	Walk
15	01	5524	5524.	Walk / wander
	03	12550	12550.	Walk/wander
	09	7875	7875.	Walk/wander
	14	9003	9003.	Walk/wander
16	15	471	471.	Walk
	16	510	510.	Walk
	21	312	312.	Walk
	22	307	307.	Walk
	31	424	424.	Walk
	32	580	580.	Walk
	47	416	416.	Walk
	58	342	342.	Walk
26	01	833	833.	Walk
27	01	1033	1033.	Walk
29	01	1316	1316.	Walk
32	01	482	482.	Walk
	02	434	434.	Walk
36	01	557	557.	Walk on uneven terrain
	04	3726	3726.	Walk on uneven terrain
	05	4196	4196.	Walk on uneven terrain
	06	3896	3896.	Walk on uneven terrain
	07	3772	3772.	Walk on uneven terrain
	08	3714	3714.	Walk on uneven terrain
	10	3832	3832.	Walk on uneven terrain
	11	4168	4168.	Walk on uneven terrain
	12	4585	4585.	Walk on uneven terrain
	13	4247	4247.	Walk on uneven terrain
	14	4146	4146.	Walk on uneven terrain
	15	4164	4164.	Walk on uneven terrain
	16	4395	4395.	Walk on uneven terrain
	17	4359	4359.	Walk on uneven terrain
	18	4304	4304.	Walk on uneven terrain
	19	4193	4193.	Walk on uneven terrain
	20	4090	4090.	Walk on uneven terrain
	21	4030	4030.	Walk on uneven terrain
	22	4312	4312.	Walk on uneven terrain
	23	4392	4392.	Walk on uneven terrain
	24	4153	4153.	Walk on uneven terrain
	25	4685	4685.	Walk on uneven terrain
	26	4237	4237.	Walk on uneven terrain
	27	4247	4247.	Walk on uneven terrain
	28	2391	2391.	Walk on uneven terrain
	29	4469	4469.	Walk on uneven terrain
	30	4414	4414.	Walk on uneven terrain
	31	3736	3736.	Walk on uneven terrain
	32	4468	4468.	Walk on uneven terrain
	33	4311	4311.	Walk on uneven terrain
	34	4328	4328.	Walk on uneven terrain
	35	4341	4341.	Walk on uneven terrain
	36	4792	4792.	Walk on uneven terrain
37	01	511	511.	Slow walk
38	01	352	352.	Walk
	02	420	420.	Walk
39	01	378	378.	Walk
	02	400	400.	Walk
	03	407	407.	Walk
	04	410	410.	Walk
	05	400	400.	Walk
	06	368	368.	Walk
	07	367	367.	Walk
	08	350	350.	Walk
	10	395	395.	Walk
	11	391	391.	Walk
	12	427	427.	Walk
	13	378	378.	Walk
	14	399	399.	Walk
43	01	421	421.	Walk
45	01	456	456.	Walk
46	01	616	616.	Walk
49	01	652	652.	Walk
55	01	530	530.	Walk
69	01	469	469.	Walk forward
	02	343	343.	Walk forward
	03	430	430.	Walk forward
	04	426	426.	Walk forward
	05	453	453.	Walk forward
81	02	544	544.	Walk forward
	03	1076	1076.	Walk
	17	916	916.	Walk forward
91	04	2162	2162.	Walk
	10	3175	3175.	Slow walk
	17	1520	1520.	Quick walk
	22	2106	2106.	Casual quick walk
	27	2894	2894.	Slow walk
	29	2181	2181.	Walk
	31	1992	1992.	Walk
	34	2208	2208.	Walk
	57	1177	1177.	Walk forward
93	07	236	236.	Casual walk
103	07	236	236.	Casual walk
104	19	921	921.	Casual walk
	35	1074	1074.	Slow walk
	356	2315	2315.	Slow walk
105	03	1865	1865.	Walk
	10	3175	3175.	Slow walk
	17	1520	1520.	Quick walk
	22	2106	2106.	Casual quick walk
	27	2894	2894.	Slow walk
	29	2181	2181.	Walk
	57	1177	1177.	Walk forward
111	34	1837	1837.	Walk
	35	1503	1503.	Walk
113	25	689	689.	Walk
114	13	3132	3132.	Walk
	14	5854	5854.	Walk
	15	1384	1384.	Walk
120	19	12792	12792.	Slow walk
	20	10735	10735.	Walk
132	17	268	268.	Walk fast
	18	425	425.	Walk fast
	19	271	271.	Walk fast
	20	342	342.	Walk fast
	21	354	354.	Walk fast
	22	371	371.	Walk fast
	45	1531	1531.	Walk slow
	46	1223	1223.	Walk slow
	47	1510	1510.	Walk slow
	48	1625	1625.	Walk slow
	49	1748	1748.	Walk slow
	50	2139	2139.	Walk slow
133	21	786	786.	Walk
	22	759	759.	Walk
	23	848	848.	Walk
136	24	1075	1075.	Walk
137	29	1128	1128.	Walk
139	28	2970	2970.	Walk
	30	1261	1261.	Walk
141	19	1193	1193.	Walk
	25	614	614.	Walk
143	32	780	780.	Walk
144	33	4688	4688.	Walk

**Table A7:** IMU configurations used during the recording of dataset **B**.

	Placement on Segment				Alignment (See Figure A1)
#	Upper Leg	Lower Leg	Foot	Pelvis	
1	anterior	anterior	dorsal	posterior	0
2	anterior	anterior	dorsal	posterior	1
3	anterior	anterior	dorsal	posterior	2
4	lateral	lateral	dorsal	posterior	0
5	lateral	lateral	dorsal	posterior	1
6	lateral	lateral	dorsal	posterior	2
7	anterior	medial	dorsal	posterior	0
8	anterior	medial	dorsal	posterior	1
9	anterior	medial	dorsal	posterior	2

## Appendix D. Cross Validation Results on Dataset A

**Table A8:** Results of the architecture cross validation for the I2S assignment problem based on dataset **A**.

	Accuracy in %		
	CNN	RNN	CNN and RNN
Assignment	96.00	98.43	100.00

**Table A9:** Results of the architecture cross validation for the I2S alignment problem based on dataset **A**: mean angle errors over all axes and windows for each segment.

	Mean Angle Errors $\left[{}^{\circ }\right]$		
	**CNN**	**RNN**	**CNN and RNN**
LeftFoot	4.201	0.796	0.623
LeftLowerLeg	2.955	0.736	0.252
LeftUpperLeg	0.770	0.729	0.427
Pelvis	1.503	1.347	1.309
RightFoot	2.670	1.336	0.800
RightLowerLeg	5.649	1.329	0.990
RightUpperLeg	1.407	3.589	0.822
**Mean**	**2.736**	**1.405**	**0.795**

## Appendix E. Evaluation Results for the Final Models

Figure A2, Figure A3, Figure A4, Figure A5, Figure A6, Figure A7, Figure A8, Figure A9, Figure A10 and Figure A11 show the evaluation results in terms of confusion matrices for the I2S assignment problem based on the final model. Each confusion matrix corresponds to one (of ten) combination(s) of test person (dataset **B** or **C**) and IMU configuration (cf. Table A7 and Figure 8). Table A10 shows the evaluation results for the I2S alignment problem.

**Table A10:** I2S alignment problem, final model, all test persons and IMU configurations: median, mean and maximum angle errors around the three body segment axes.

Segment	$\mathrm{ax}0[{}^{\circ }]$			$\mathrm{ax}1[{}^{\circ }]$			$\mathrm{ax}2[{}^{\circ }]$		
	Med	Mean	Max	Med	Mean	Max	Med	Mean	Max
LeftFoot	1.48	13.71	165.06	2.38	6.99	44.70	4.02	12.21	155.23
LeftLowerLeg	4.20	28.59	178.40	3.16	14.22	75.82	7.04	17.95	116.93
LeftUpperLeg	1.35	14.35	171.09	0.88	8.40	81.11	1.51	10.13	96.67
Pelvis	1.42	13.53	180.00	2.07	7.28	88.45	0.65	12.89	179.98
RightFoot	2.96	16.12	127.48	1.35	5.23	72.81	2.39	11.42	157.63
RightLowerLeg	3.61	31.21	179.97	7.63	10.92	52.61	5.13	24.49	179.79
RightUpperLeg	3.54	28.55	178.10	1.30	9.42	77.72	3.02	21.79	179.64
**Average**	**2.65**	**20.86**	**168.58**	**2.68**	**8.92**	**70.46**	**3.39**	**15.84**	**152.26**
